# Supplementary material for: Hybrid-control arm construction using historical trial data for an early-phase, randomized controlled trial in metastatic colorectal cancer
Source: Commun Med (Lond). 2022 Jul 15;2:90. doi: 10.1038/s43856-022-00155-y (PMC9287310; doi:10.1038/s43856-022-00155-y)
Supplement: Supplementary file 1 — Description of Additional Supplementary Files [file 43856_2022_155_MOESM1_ESM.pdf]

Description of Additional Supplementary Files

File Name: Supplementary Data 1

Description: Comparison of Trial Eligibility Criteria Between the IMblaze370 and the MORPHEUS mCRC
