# Supplementary material for: Hybrid-control arm construction using historical trial data for an early-phase, randomized controlled trial in metastatic colorectal cancer
Source: Commun Med (Lond). 2022 Jul 15;2:90. doi: 10.1038/s43856-022-00155-y (PMC9287310; doi:10.1038/s43856-022-00155-y)
Supplement: Supplementary file 4 — Supplementary Information [file 43856_2022_155_MOESM4_ESM.pdf]

Supplementary Online Content

Li C, Ferro A, Mhatre SK, Lu D, Lawrance M, Li X, Li S, Allen S, Desai J, Fakih M, Cecchini M, Pedersen KS, Kim TY, Reyes-Rivera I, Segal N, Lenain C. Hybrid-control arm construction using historical trial data for an early-phase, randomized controlled trial in metastatic colorectal cancer. *Communications Medicine*

This supplementary material has been provided by the authors to give readers additional information about their work.

Supplementary Figure 1. Study Design of the MORPHEUS-CRC Study
Supplementary Figure 2. Prior and posterior distributions of β coefficients in the DCR, PFS and OS models
Supplementary Figure 3. CONSORT diagram
Supplementary Figure 4. Comparison of Standardized Mean Differences (SMD) Before and After Weighting
Supplementary Figure 5. Kaplan-Meier Curves for Progression-Free and Overall Survival with Standardized Mortality Ratio Weighting.

Supplementary Data File 1. Comparison of Trial Eligibility Criteria Between the IMblaze370 and the MORPHEUS mCRC

Supplementary Table 1. Sensitivity analyses with different prior distributions for τ
Supplementary Table 2. MORPHEUS-CRC Safety Summary
Supplementary Table 3. MORPHEUS-CRC Treatment-Related Adverse Events
Supplementary Table 4. DCR Estimation in the Original Data Sets without Weighting

Supplementary Notes
Supplementary Note 1. Rationale for choosing the SMRW method over the IPTW method for the purpose of propensity weighting
Supplementary Note 2. Inclusion and exclusion criteria for the experimental arm (atezolizumab + isatuximab) and the control arm (regorafenib) of the MORPHEUS-CRC study.
Supplementary Note 3. A simulation study to compare the results of the frequentist method and the Bayesian method in terms of their statistical properties, including variance, MSE and 95% coverage.
Supplementary Note 4. List of Institutional Review Boards (IRBs) that approved the MORPHEUS-CRC trial from the study sites that enrolled patients in the arms described in the manuscript.

Supplement References

Supplementary Figure 1. Study Design of the MORPHEUS-CRC Study

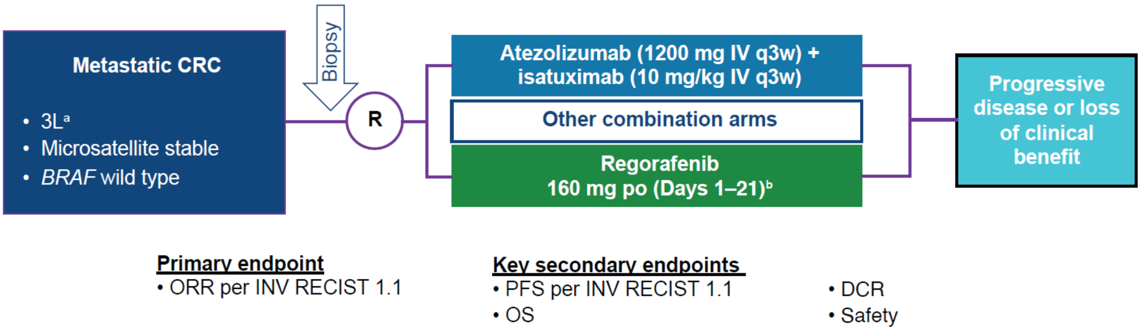

[a] Prior treatment with fluoropyrimidine, oxaliplatin or irinotecan-containing chemotherapy plus a biologic agent.
[b] Dose escalation to 160 mg during Cycle 1 was allowed per institutional guidelines.

Abbreviations: 3L, third line; *BRAF*, B-Raf proto-oncogene, serine/threonine kinase; CRC, colorectal cancer; DCR, disease control rate; IV, intravenously; INV, investigator-assessed; ORR, objective response rate; OS, overall survival; PFS, progression-free survival; po, orally; q3w, every 3 weeks; R, randomized; RECIST, Response Evaluation Criteria in Solid Tumors.

Supplementary Figure 2. Prior (blue) and posterior (red) distributions of β coefficients in the a) DCR, b) PFS and c) OS models. The priors all follow standard normal distributions, and the posterior distributions look normal as well, yet the posterior means are shifted a bit to the right compared to the prior means and the variances become smaller.

a) DCR

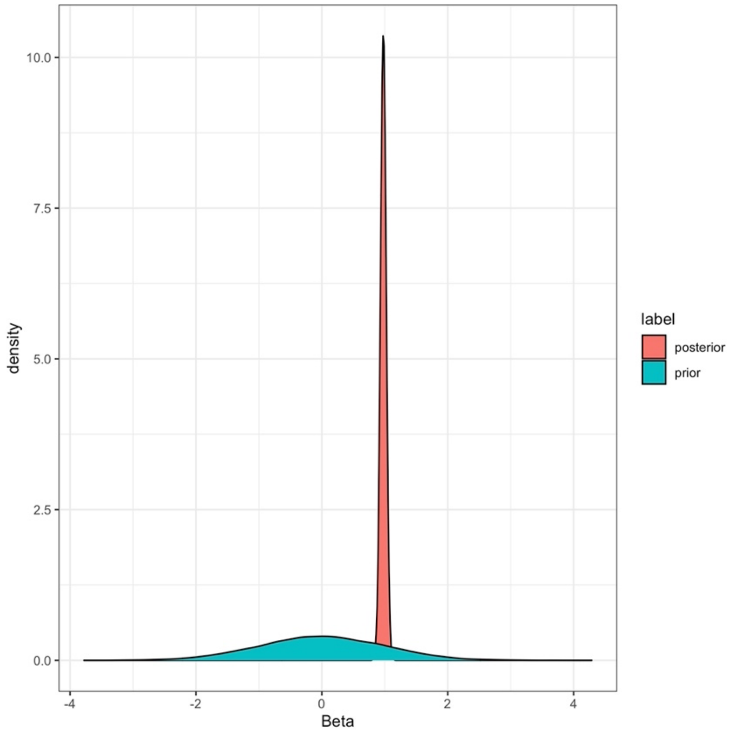

b) PFS

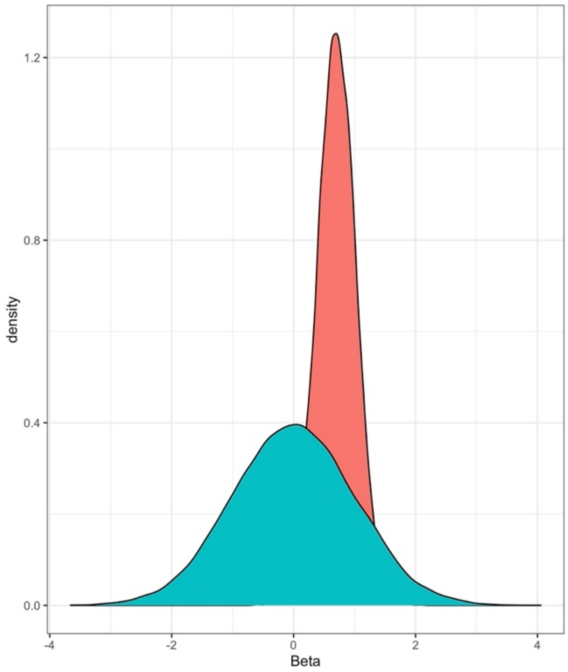

c) OS

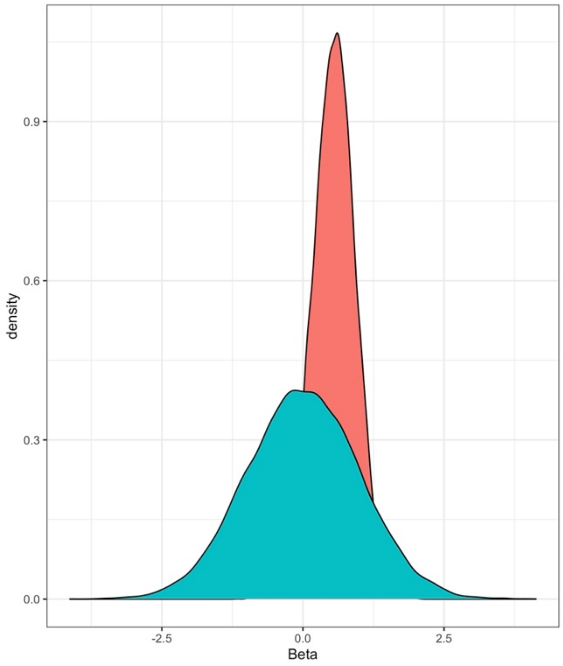

Supplementary Figure 3. CONSORT 2010 Flow diagram (MORPHEUS CRC).

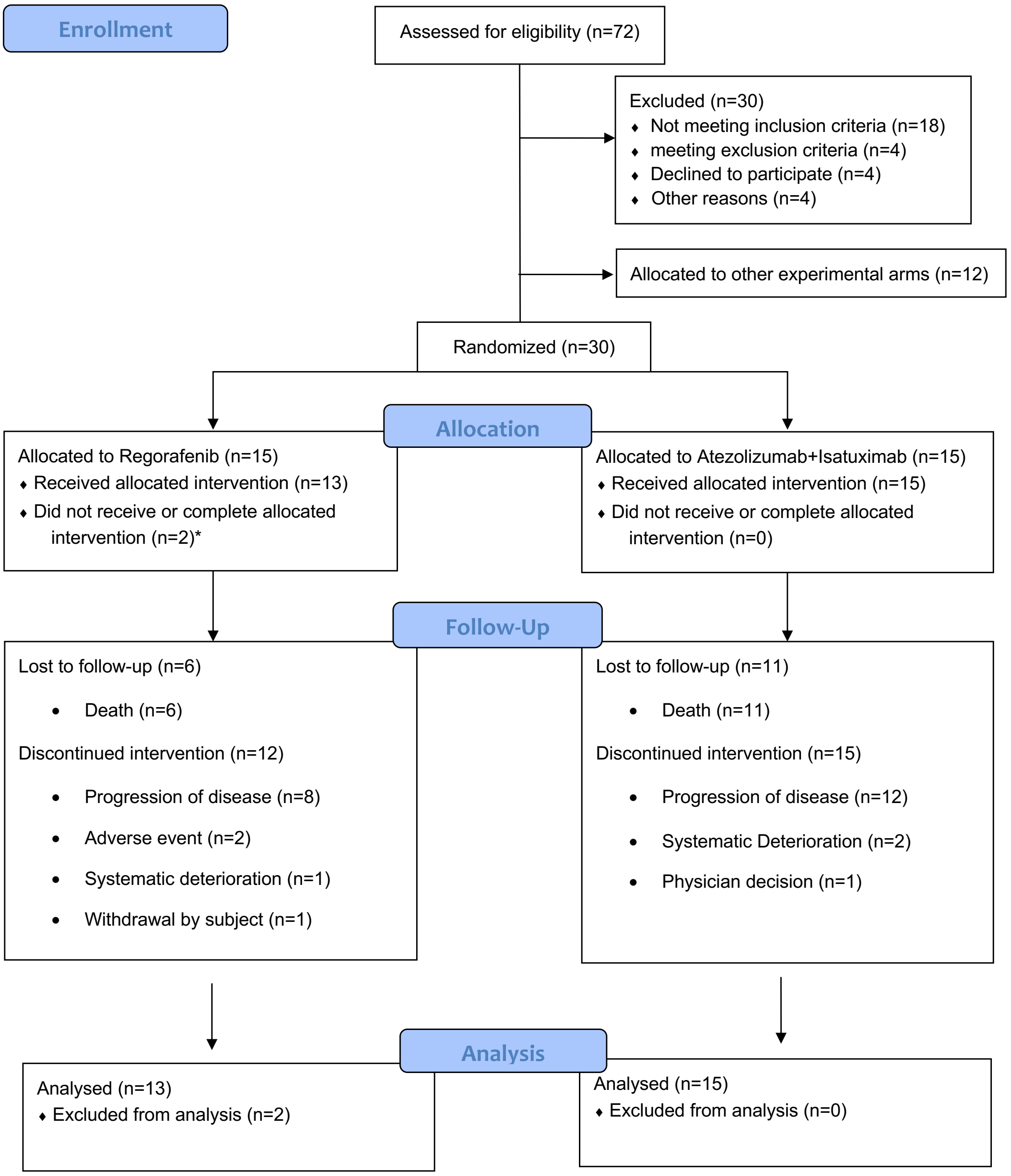

**Two patients left the study after randomization before receiving a dose of treatment in the control arm, and we define the efficacy evaluable population as "all patients who receive at least one dose of each drug for their assigned treatment regimen".*

Supplementary Figure 4. Comparison of Standardized Mean Differences (SMD) Before and After Weighting

Predefined prognostic factors are adjusted using the propensity score weighting method. The plot shows balance (SMD <0.25) achieved for each variable after the weighting.

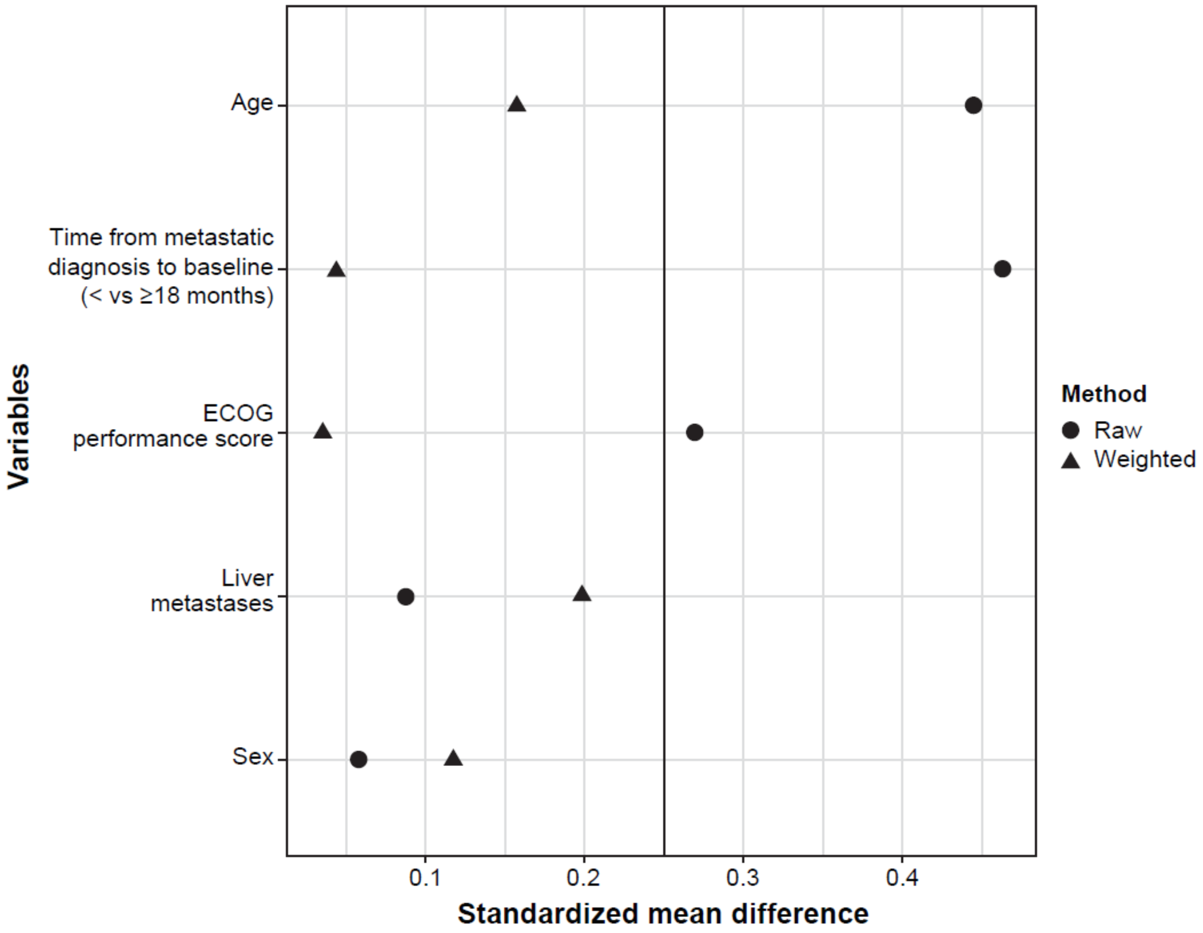

Abbreviations: ECOG, Eastern Cooperative Oncology Group; SMD, standardized mean difference.

Supplementary Figure 5. Kaplan-Meier Curves for Progression-Free (A) and Overall Survival (B) with Standardized Mortality Ratio Weighting

Survival curves were shown by treatment arms. Median survival time was indicated via right-angle folded black dash lines connecting 0.50 on the y axis to the corresponding months on the x axis. Absolute numbers and percentages of patients at risk over time were shown in the table below plots. Note that the starting percentages in the external-control and hybrid-control cohorts are not 100, reflecting that these are weighted pseudo-populations.

A

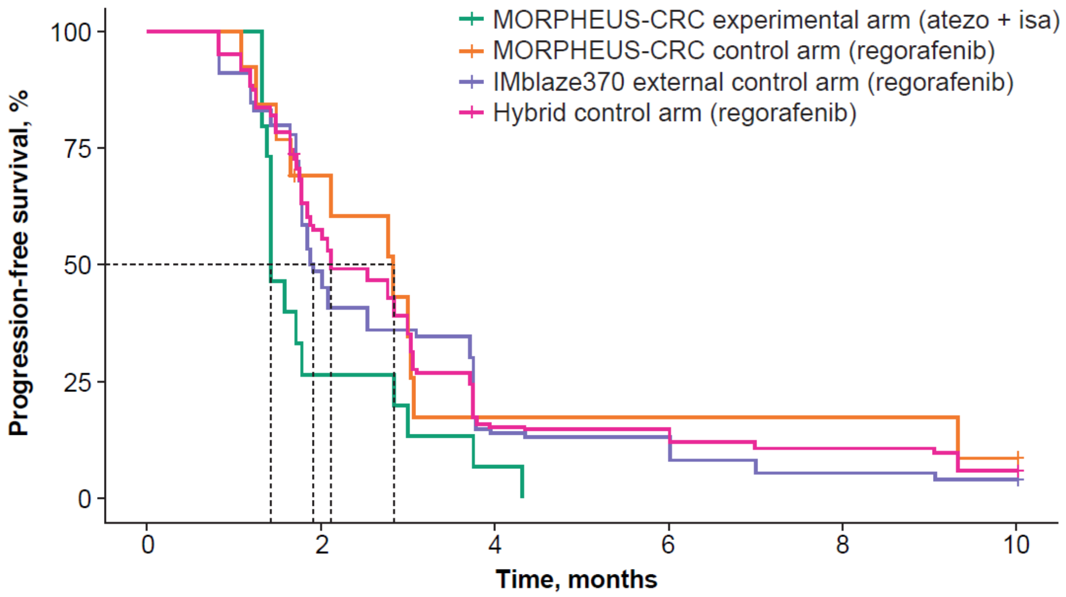

15 (100)	4 (27)	1 (7)	0 (0)	0 (0)	0 (0)
13 (100)	8 (62)	2 (15)	2 (15)	2 (15)	1 (8)
15 (52)	7 (25)	2 (7)	2 (7)	1 (3)	1 (2)
28 (67)	15 (37)	4 (10)	4 (10)	3 (7)	2 (4)

B

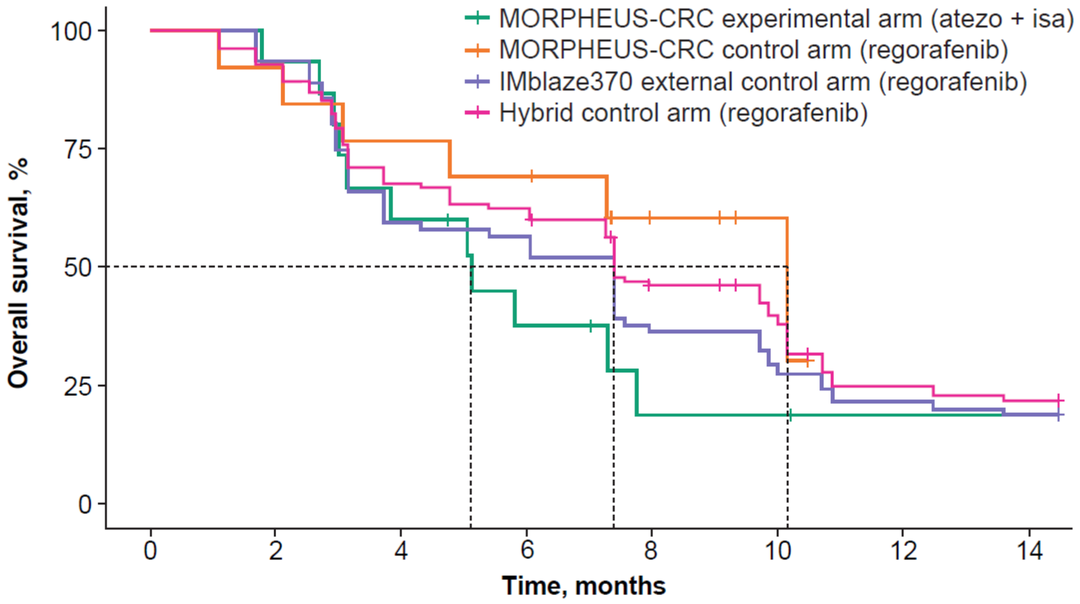

15 (100)	14 (93)	9 (60)	5 (33)	2 (13)	2 (13)	1 (7)	1 (7)
13 (100)	12 (92)	10 (77)	9 (69)	4 (31)	2 (15)	0 (0)	0 (0)
15 (52)	14 (49)	9 (31)	8 (29)	5 (19)	4 (15)	3 (11)	3 (10)
28 (67)	26 (62)	19 (45)	17 (42)	9 (23)	6 (15)	3 (8)	3 (7)

**Arm**	**Median Survival (95% CI), months**	
	**PFS**	**OS**
MORPHEUS-CRC CC (regorafenib)	2.83 (1.64-3.06)	10.15 (4.76-NE)
MORPHEUS-CRC EXP (atezo + isa)	1.41 (1.41-1.77)	5.13 (3.12-7.75)
EC (regorafenib)	1.91 (1.74-3.75)	7.39 (3.15-10.71)
HC (regorafenib)	2.10 (1.77-3.09)	7.39 (4.76-10.87)

Abbreviations: CC, concurrent control; EC, external control; EXP, experimental; HC, hybrid control; OS, overall survival; PD, progressive disease; PFS, progression-free survival; SD, stable disease; NE, not estimable.

Supplementary Data File 1. Comparison of Trial Eligibility Criteria Between the IMblaze370 and the MORPHEUS-mCRC Trials.

Abbreviations: 3L, third-line; *BRAF*, B-Raf proto-oncogene, serine/threonine kinase; CBA, cerebrovascular accidents; CNS, central nervous system; DCR, disease control rate; MSS, microsatellite stable; MSI-H, microsatellite instability–high; MAPK/ERKi, mitogen-activated protein kinase/extracellular signal-regulated kinase inhibitor; mCRC, metastatic colorectal cancer; PD, progressive disease.

Supplementary Table 1. Treatment effect estimates of DCR, PFS and OS for different prior distributions.

	τ ~ Gamma(1,1)	τ ~ Gamma(1,0.1)	τ ~ Gamma(1,0.01)
DCR (OR [95% CI])	0.82 [0.19, 2.91]	1.08 [0.19, 3.56]	1.18 [0.21, 3.86]
	τ ~ half-Cauchy(0, 25)	τ ~ half-Cauchy(0, 50)	τ ~ half-Cauchy(0, 100)
PFS (HR [95% CI])	2.10 [1.03, 3.74]	2.10 [1.05, 3.77]	2.11 [1.04, 3.70]
OS (HR [95% CI])	1.88 [0.82, 3.60]	1.88 [0.83, 3.57]	1.90 [0.85, 3.57]

Supplementary Table 2. MORPHEUS-CRC Safety Summary
Clinical cutoff: 3 March 2020.

	**Atezolizumab + isatuximab (n = 15)**	**Regorafenib (n = 13)**
All-grade AE, any cause, No. (%)	15 (100)	13 (100)
Treatment-related all-grade AE[a]	13 (87)	12 (92)
Grade 3-4 AE, No. (%)	7 (47)	8 (62)
Treatment-related Grade 3-4 AE	2 (13)	8 (62)
Serious AE, No. (%)	5 (33)	3 (23)
Treatment-related serious AE	0	1 (8)
Grade 5 AE, No. (%)	0	1 (8)
AE leading to withdrawal from treatment, No. (%)	0	2 (15)
Treatment-related AE leading to withdrawal from treatment	0	1 (8)
AE leading to dose modification/interruption, No. (%)	2 (13)	8 (62)
Treatment-related AE leading to dose modification/interruption	0	7 (54)

Abbreviations: AE, adverse event.

Supplementary Table 3. MORPHEUS-CRC Treatment-Related Adverse Events
Clinical cutoff: 3 March 2020.

	**Atezolizumab + isatuximab (n = 15)**	**Regorafenib (n = 13)**
Infusion-related reaction	11 (73)	0
Nausea	4 (27)	3 (23)
Fatigue	3 (20)	4 (31)
Diarrhea	2 (13)	5 (39)
Decreased appetite	1 (7)	4 (31)
Palmar-plantar erythrodysesthesia syndrome	0	8 (62)
Pain in extremity	0	4 (31)
Dysphonia	0	3 (23)

Supplementary Table 4. DCR Estimation in the Original Data Sets without Weighting. [a]Criteria for disease control is either response and/or stable disease or better for at least 12 weeks. [b]Patients were classified as achieving stable disease or progressive disease if assessment was at least 12 weeks from randomization. [c]Patients were classified as missing if no post-baseline response at the corresponding assessments were available.

	EC (n = 28)	Ctrl (n = 13)	Exp (n = 15)
DCR, No. (%)[a]	6 (21.4)	2 (15.4)	2 (13.3)
SD, No. (%)[b]	6 (21.4)	2 (15.4)	2 (13.3)
PD, No. (%)[b]	17 (60.7)	9 (69.2)	11 (73.3)
Responders, No. (%)	0 (0.0)	0 (0.0)	0 (0.0)
Unknown, No. (%)[c]	5 (17.9)	2 (15.4)	2 (13.3)

Abbreviations: Ctrl, control; DCR, disease control rate; EC, external control; Exp, experimental; PD, progressive disease; SD, stable disease.

Supplementary Note 1. Rationale for choosing the SMRW method over the IPTW method for the purpose of propensity weighting

We chose the SMRW over the IPTW as the propensity score weighting method due to a couple of considerations.

Firstly, different approaches can result in different estimands of the treatment effects (ICH E9(R1) Addendum). For the IPTW, the treatment effect is the population average treatment effect (ATE), hence the results can be generalised to the entire population from which the observed samples are representative of, whereas the SMRW method results in an estimate of the average treatment effect in the treated (ATT), therefore focusing on the patients who would receive the intervention treatment. In randomised controlled trials, ATT and ATE are the same as we assume baseline characteristics and treatment effects in the control and the treated groups are comparable, but in studies that contain non-randomisation components, ATT and ATE estimates can be different, as they target different populations. The choice depends on varied study interests, in medical studies, the ATT is typically used because clinicians often care more about the causal effect of drugs for a targeted group of patients who would potentially receive the intervention drugs.

Secondly, the propensity score method was originally developed to build external controls in single arm trials, hence there are only two arms (the trial experimental arm and the external control arm) to be weighted. However, in a hybrid control study, we have three arms, the experimental and control arms from the MORPHEUS trial and the externally derived control arm from the historical IMblaze370 trial. In our propensity score model (i.e., the logistic regression), the externally derived control arm and the experimental arm are used as the treatment indicator (the dependent variable), the internal control arm is not used in the model, but also must also be weighted. By using the SMRW, both the internal control and experimental arms are given a weight of 1, under the assumption that few confounding effects exist after randomisation, thereby solving the problem of unknown weights for the internal control arm.

Supplementary Note 2. Inclusion and exclusion criteria for the experimental arm (atezolizumab + isatuximab) and the control arm (regorafenib) of the MORPHEUS-CRC study

Inclusion Criteria:

1. Eastern Cooperative Oncology Group (ECOG) Performance Status of 0 or 1
2. Life expectancy ≥ 3 months, as determined by the investigator
3. Histologically confirmed adenocarcinoma originating from the colon or rectum
4. Metastatic disease not amenable to local treatment
5. Disease progression during or following not more than two separate lines of treatment for metastatic colorectal cancer (mCRC) that consisted of fluoropyrimidine-, oxaliplatin-, and irinotecan-containing chemotherapy in combination with a biologic agent
6. Measurable disease (at least one target lesion) according to RECIST v1.1
7. Adequate hematologic and end-organ function obtained within 14 days prior to initiation of study treatment

Exclusion Criteria:

1. High microsatellite instability (MSI-H) tumour
2. Presence of BRAFV600E mutation
3. Prior treatment with any of the protocol-specified study treatments
4. Prior treatment with T-cell co-stimulating or immune checkpoint blockade therapies including anti-CTLA-4, anti-PD-1, and anti-PD-L1 therapeutic antibodies
5. Biologic treatment within 2 weeks prior to initiation of study treatment, or other systemic treatment for CRC within 2 weeks or 5 half-lives of the drug (whichever is shorter) prior to initiation of study treatment
6. Treatment with investigational therapy within 28 days prior to initiation of study treatment

Eligibility only for the control arm

7. Prior allogeneic stem cell or solid organ transplantation
8. Treatment with systemic immunostimulatory agents within 4 weeks or 5 half-lives of the drug (whichever is longer) prior to the initiation of study treatment
9. Treatment with systemic immunosuppressive medication within 2 weeks prior to initiation of study treatment, or anticipation of need for systemic immunosuppressant medication during study treatment
10. Treatment with a live, attenuated vaccine within 4 weeks prior to initiation of study treatment, or anticipation of need for such a vaccine during atezolizumab treatment or within 5 months after the last dose of atezolizumab
11. Current treatment with anti-viral therapy for HBV
12. Uncontrolled pleural effusion, pericardial effusion, ascites requiring recurrent drainage procedures (once monthly or more frequently), or tumor related pain,
13. Uncontrolled or symptomatic hypercalcemia (ionized calcium >1.5 mmol/L, calcium >12 mg/dL, or corrected serum calcium >ULN)
14. Symptomatic, untreated, or actively progressing CNS metastases
15. History of leptomeningeal disease
16. Active or history of autoimmune disease or immune deficiency
17. History of idiopathic pulmonary fibrosis, organizing pneumonia, drug-induced pneumonitis, or idiopathic pneumonitis, or evidence of active pneumonitis on screening chest computed tomography (CT) scan
18. History of malignancy other than CRC within 2 years prior to screening, except for malignancies with a negligible risk of metastasis or death
19. Active tuberculosis
20. Severe infection within 4 weeks prior to initiation of study treatment
21. Significant cardiovascular disease

22. Grade ≥3 haemorrhage or bleeding event within 28 days prior to initiation of study treatment
23. Major surgical procedure, other than for diagnosis, within 4 weeks prior to initiation of study treatment, or anticipation of need for a major surgical procedure during the study
24. History of severe allergic reactions to chimeric or humanized antibodies or fusion proteins
25. Inability to swallow medications
26. Malabsorption condition that would alter the absorption of orally administered medications
27. Evidence of inherited bleeding diathesis or significant coagulopathy at risk of bleeding
28. Urine dipstick ≥ 2+ protein or ≥ 3.5 g of protein in a 24-hour urine collection

EC specific to isatuximab:
1. Prior treatment with an agent that blocks CD38
2. Known intolerance to any of the drugs and their equivalents required for isatuximab premedication

Supplementary Note 3. A simulation study to compare the results of the frequentist method and the Bayesian method in terms of their statistical properties, including variance, MSE and 95% coverage

we performed a simulation study with 1,000 iterations with known parameters and compared the results of the frequentist method and the Bayesian method in terms of their statistical properties, including variance, MSE and 95% coverage.

We assumed a historical control sample size of 30 and a trial sample size of 30 with a probability of 0.535 (observed fraction) for being assigned to the experimental arm. Covariates included age, sex, presence of liver metastases, time from metastatic diagnosis to baseline (> vs ≤18 months) and ECOG values. Ages in the historical control group and the trial group were assumed to follow normal distributions with mean and standard deviation equivalent to those in the corresponding groups. Other covariates were all binary types and followed binomial distributions with probabilities equivalent to those estimated in the real dataset.

We assumed a Weibull baseline hazard function $h_0(t)=\lambda\rho t^{\rho-1}$ with a shape ρ=1.2 and a scale λ=0.1; the cumulative baseline hazard function can then be expressed as $H_0(t)= \lambda t^{\rho}$. To generate survival time for each patient, we randomly drew v from a uniform distribution U(0,1), and made an inverse transformation of $H_0(t)$: $H_0^{-1}(t)=(t/\lambda)^{1/\rho}$, then survival time t can be computed as $(-\log(v)/\lambda\exp(\mathbf{x}'\beta))^{1/\rho}$, where $\mathbf{x}'$ is a matrix of covariates and β is a vector of coefficients estimated from Cox proportional-hazards model in real dataset. Censoring time c was assumed to follow an exponential distribution of rate = ¼, then the censored survival time was defined as the smaller value of the survival time t and the censoring time c.

Results are shown below. The Bayesian method shows better precision (smaller variance) and accuracy (less bias) than the frequentist method in this simulation setting, where the sample size is very small, e.g. in the early phase trial setting.

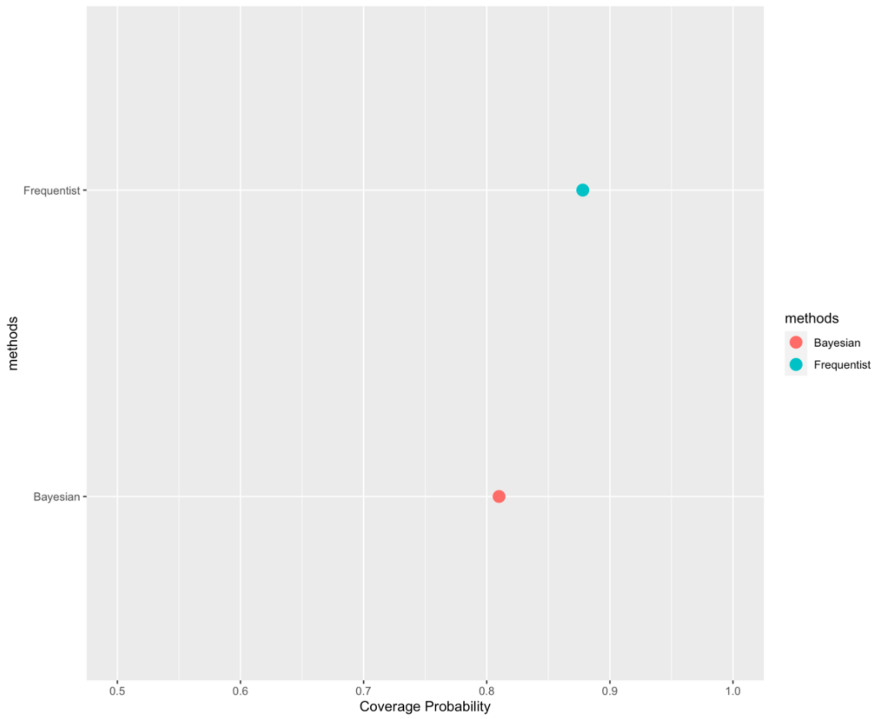

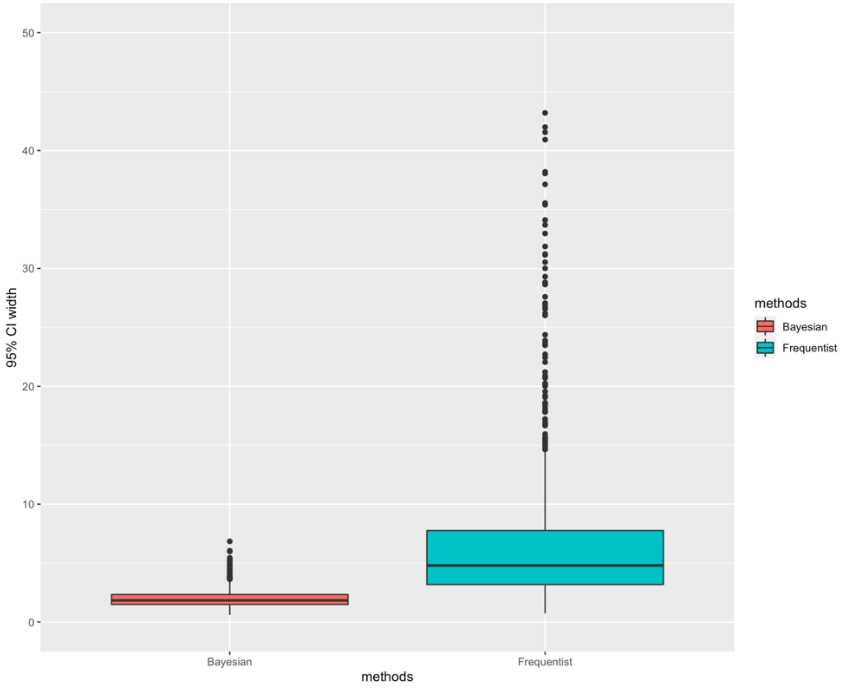
50
40
30
20
10
0
95% CI width
Bayesian
Frequentist
methods
methods
Bayesian
Frequentist

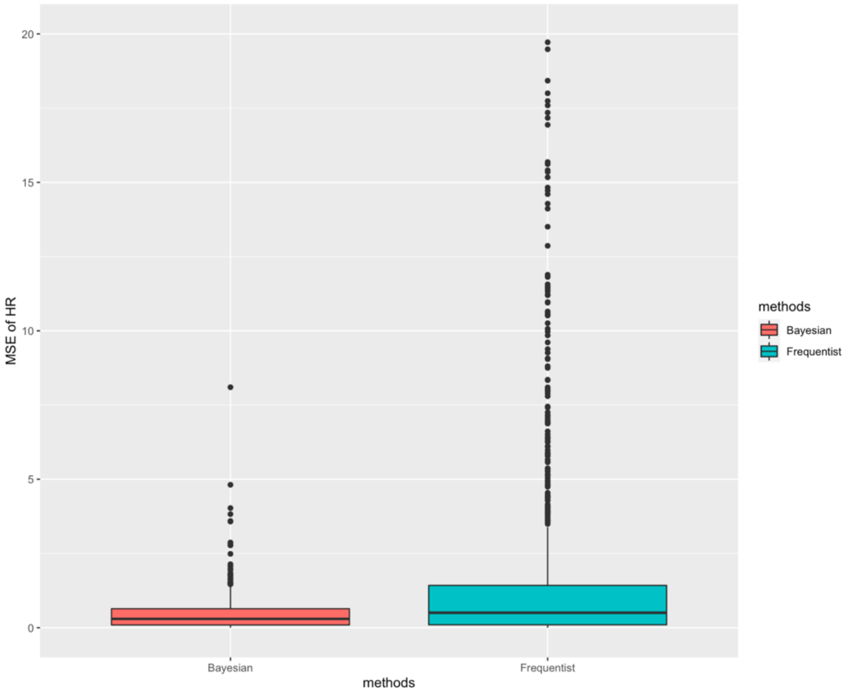
20
15
10
5
0
MSE of HR
Bayesian
Frequentist
methods
methods
Bayesian
Frequentist

Supplementary Note 4. List of Institutional Review Boards (IRBs) that approved the MORPHEUS-CRC trial from the study sites that enrolled patients in the arms described in the manuscript.

**Protocol Country**	**Site Account**	**IRB Name**	**IRB number**	**First Name**	**Country**
United States	Memorial Sloan-Kettering Cancer Center	MSKCC	IRB0000273	IRB	USA
United States	Yale School Of Medicine	WIRB	IRB00000533	IRB	USA
United States	City of Hope - Duarte	WIRB	IRB00000533	IRB	USA
United States	Washington University School of Medicine in St. Louis	WIRB	(WIRB) IRB00000533	IRB	USA
Australia	Peter MacCallum Cancer Centre	Peter MacCallum Cancer Centre Ethics Committee	EC00235	HREC	AUS
Korea, South	Seoul National University Hospital	IRB of Seoul National University Hospital	H-1807-181-963	IRB	KOR
Korea, South	Asan Medical Center	IRB of Asan Medical Center	2018-1000	IRB	KOR
France	Gustave Roussy	COMITE DE PROTECTION DES PERSONNES	No number available (classified by Region) - Comité de Protection des Personnes Sud Est V. EC accredited by MOH	Sud Est V	FRA

Supplementary References

1. Bekaii-Saab TS, Ou FS, Ahn DH, et al. Regorafenib dose-optimisation in patients with refractory metastatic colorectal cancer (ReDOS): a randomised, multicentre, open-label, phase 2 study. *Lancet Oncol.* 2019;20(8):1070-1082. doi: 10.1016/S1470-2045(19)30272-4.
