# Supplementary material for: Hybrid-control arm construction using historical trial data for an early-phase, randomized controlled trial in metastatic colorectal cancer
Source: Commun Med (Lond). 2022 Jul 15;2:90. doi: 10.1038/s43856-022-00155-y (PMC9287310; doi:10.1038/s43856-022-00155-y)
Supplement: Supplementary file 5 — Reporting Summary [file 43856_2022_155_MOESM5_ESM.pdf]

nature research

Corresponding author(s): Chen Li and Christelle Lenain

Last updated by author(s): Jun 3, 2022

Reporting Summary

Nature Research wishes to improve the reproducibility of the work that we publish. This form provides structure for consistency and transparency in reporting. For further information on Nature Research policies, see our Editorial Policies and the Editorial Policy Checklist.

Statistics

For all statistical analyses, confirm that the following items are present in the figure legend, table legend, main text, or Methods section.

n/a	Confirmed	
☐	☒	The exact sample size (n) for each experimental group/condition, given as a discrete number and unit of measurement
☐	☒	A statement on whether measurements were taken from distinct samples or whether the same sample was measured repeatedly
☐	☒	The statistical test(s) used AND whether they are one- or two-sided<br>*Only common tests should be described solely by name; describe more complex techniques in the Methods section.*
☐	☒	A description of all covariates tested
☐	☒	A description of any assumptions or corrections, such as tests of normality and adjustment for multiple comparisons
☐	☒	A full description of the statistical parameters including central tendency (e.g. means) or other basic estimates (e.g. regression coefficient) AND variation (e.g. standard deviation) or associated estimates of uncertainty (e.g. confidence intervals)
☐	☒	For null hypothesis testing, the test statistic (e.g. F, t, r) with confidence intervals, effect sizes, degrees of freedom and P value noted<br>*Give P values as exact values whenever suitable.*
☐	☒	For Bayesian analysis, information on the choice of priors and Markov chain Monte Carlo settings
☐	☒	For hierarchical and complex designs, identification of the appropriate level for tests and full reporting of outcomes
☐	☒	Estimates of effect sizes (e.g. Cohen's d, Pearson's r), indicating how they were calculated

Our web collection on statistics for biologists contains articles on many of the points above.

Software and code

Policy information about availability of computer code

Data collection	no software was used
Data analysis	RStudio version 1.3.0 and R version 3.6.3.

For manuscripts utilizing custom algorithms or software that are central to the research but not yet described in published literature, software must be made available to editors and reviewers. We strongly encourage code deposition in a community repository (e.g. GitHub). See the Nature Research guidelines for submitting code & software for further information.

Data

Policy information about availability of data

All manuscripts must include a data availability statement. This statement should provide the following information, where applicable:

- Accession codes, unique identifiers, or web links for publicly available datasets
- A list of figures that have associated raw data
- A description of any restrictions on data availability

Qualified researchers engaged in rigorous, independent scientific research may request access to individual patient-level data upon request through https://www.roche.com/innovation/process/clinical-trials/data-sharing/request. Data from individual patients can be requested 18 months after relevant clinical studies being approved by the regulatory authorities or will not be developed further. Requests outside this scope will be considered on a case-by-case basis through enquiries via the https://vivli.org site. Further details on data sharing and how to request access to related clinical study documents are available at https://www.roche.com/innovation/process/clinical-trials/data-sharing/. Information on the clinical trials can be found on clinicaltrials.gov, or the Imblaze370 trial via https://pubmed.ncbi.nlm.nih.gov/31003911/.

Field-specific reporting

Please select the one below that is the best fit for your research. If you are not sure, read the appropriate sections before making your selection.

☒ Life sciences ☐ Behavioural & social sciences ☐ Ecological, evolutionary & environmental sciences

For a reference copy of the document with all sections, see nature.com/documents/nr-reporting-summary-flat.pdf

Life sciences study design

All studies must disclose on these points even when the disclosure is negative.

Sample size

This study is not designed to make explicit power and type I error considerations for a hypothesis test. Instead, the trial study is designed to obtain preliminary data on immunotherapy-based treatment combinations when administered to patients with metastatic colorectal cancer who experienced disease progression during or following two lines of treatment for metastatic colorectal cancer that consisted of fluoropyrimidine-, oxaliplatin-, or irinotecan-containing chemotherapy in combination with a biologic agent (e.g., bevacizumab, cetuximab), given in combination as two separate lines of therapy (in either order). Approximately 15 patients were randomly allocated to control or each experimental arms during the study.

The sample size for the hybrid control design was derived via a step-wise mapping of trial inclusion / exclusion criteria onto the external control cohort -- the historical trial IMblaze370 control arm.

Data exclusions

No data was excluded from the analyses.

Replication

No reproduction studies for clinical practice of this trial have been conducted yet, due to nature of early phase trials and recent complement of the study operation. Further assessment for clinical trial reproducibility may be performed at a later phase.

Randomization

For Stage 1, this study employed a permuted-block randomization method with dynamically changing randomization ratios to account for fluctuation in the number of treatment arms that were open for enrollment over the course of the study. The randomization ratio depended on the number of experimental arms that were open for enrollment (e.g., if an arm was added or enrollment in an arm was suspended pending analysis of results from the preliminary phase), with the stipulation that no more than 35% of patients were randomly allocated to the control arm at any given time. Randomization took into account general inclusion and exclusion criteria.

Blinding

The investigators were not blinded to group allocation and / or analysis. This study is a Phase Ib/II, open-label, multicenter, randomized, umbrella study in patients with metastatic colorectal cancer. The study is designed with flexibility to open new treatment arms when new treatments become available, close existing treatment arms that demonstrate minimal clinical activity or unacceptable toxicity, or modify the patient population (e.g., with regard to prior anti-cancer treatment or biomarker status). By design, patients who experience disease progression, loss of clinical benefit, or unacceptable toxicity during Stage 1 may be eligible to continue treatment with a different treatment regimen.

Reporting for specific materials, systems and methods

We require information from authors about some types of materials, experimental systems and methods used in many studies. Here, indicate whether each material, system or method listed is relevant to your study. If you are not sure if a list item applies to your research, read the appropriate section before selecting a response.

Materials & experimental systems

n/a	Involved in the study	
☒	☐	Antibodies
☒	☐	Eukaryotic cell lines
☒	☐	Palaeontology and archaeology
☒	☐	Animals and other organisms
☐	☒	Human research participants
☐	☒	Clinical data
☒	☐	Dual use research of concern

Methods

n/a	Involved in the study	
☒	☐	ChIP-seq
☒	☐	Flow cytometry
☒	☐	MRI-based neuroimaging

Human research participants

Policy information about studies involving human research participants

Population characteristics

The study enrolled patients with microsatellite stable metastatic colorectal cancer who had experienced disease progression during or following two lines of treatment for metastatic colorectal cancer. The patient population average age was over 50 years, and the majority were white people recruited mainly from North America and Europe, yet about 30% of patients were non-whites recruited from Asia pacific region. The ECOG Performance Status for all patients was below 2, and over half of the patients had time from metastatic diagnosis to baseline larger than 18 months. The majority of patients also had liver

	metastases. The population showed no differences in sex and RAS mutation status.
Recruitment	Patients who met the trial inclusion / exclusion criteria were recruited.
Ethics oversight	IRB/EC

Note that full information on the approval of the study protocol must also be provided in the manuscript.

Clinical data

Policy information about clinical studies

All manuscripts should comply with the ICMJE guidelines for publication of clinical research and a completed CONSORT checklist must be included with all submissions.

Clinical trial registration	NCT03555149
Study protocol	It can be available from corresponding authors on reasonable requests, due to confidentiality of an ongoing trial.
Data collection	Data entered manually were collected via electronic data collection through use of electronic case report forms. When a study site's computerized medical record system has been in use (i.e., in lieu of original hardcopy records), the electronic record can serve as the data source document if the system has been validated in accordance with health authority requirements pertaining to computerized systems used in clinical research. Data collection was initiated within 3 days from recruitment.
Outcomes	ORR, defined as the proportion of patients with a complete response or partial response on two consecutive occasions >= 4 weeks apart, as determined by the investigator according to RECIST v1.1. DCR, defined as the proportion of patients with stable disease for >= 12 weeks or a complete or partial response, as determined by the investigator according to RECIST v1.1. PFS after randomization, defined as the time from randomization to the first occurrence of disease progression or death from any cause (whichever occurs first), as determined by the investigator according to RECIST v1.1. OS after randomization, defined as the time from randomization to death from any cause.
